# Executive functioning in matrescence and implications for perinatal depression

**DOI:** 10.3389/fpsyt.2025.1663017

**Published:** 2025-09-19

**Authors:** T. Roxana Ghadimi, Clare McCormack

**Affiliations:** Department of Child and Adolescent Psychiatry, New York University (NYU) Grossman School of Medicine, New York, NY, United States

**Keywords:** pregnancy, cognition, executive function, maternal depression, mental load

## Abstract

The perinatal period represents a time of profound neurobiological, cognitive, and emotional change. While evidence points to the neuroplasticity of matrescence as adaptive in supporting the transition to motherhood, the perinatal period also entails subjective reports of cognitive difficulty known as “mommy brain” as well as a heightened vulnerability to mental health challenges. The role of cognition in the etiology of postpartum depression is a promising area of investigation into targets for maternal mental health intervention, considering evidence that important cognitive changes occur during the perinatal period, and given that cognitive alterations are key features of mood disorders. Here we review evidence for cognitive plasticity in matrescence, with a particular focus on executive function (EF) given its overlapping significance for adaptation to parenthood, central role in managing the mental load of motherhood, and implications in mood regulation and mood disorders. We also review evidence for EF changes in perinatal depression and major depressive disorder more broadly. Despite the strong association between EF impairments and major depressive disorder, research on EF changes in perinatal depression remains limited. Understanding normative EF changes during this period is essential for better understanding the relationship between EF, perinatal depression, and the mental load of motherhood. Consideration for these cognitive, neurobiological, and psychosocial factors of matrescence is critical for addressing maternal mental health and developing interventions that support parental well-being.

## 1 Introduction

In two recent health advisories and calls to action, the U.S. Surgeon General highlighted the need to improve the physical and mental health of mothers and mothers-to-be, as well as reduce the burden of parental stress (1, 2). This reflects the seriousness of challenges facing women across the perinatal period and an urgent unmet need. Perinatal mood and anxiety disorders (PMADs) are the most common birth complication, with perinatal depression affecting approximately 1-in 7 mothers and disproportionately impacting women of color (3–5). PMADs have serious consequences for both mother and baby via associations with preterm birth, compromised parenting quality, maternal substance use and suicide, and long-term impairments in infant and child cognitive social-emotional development, behavior, and family functioning (6–8). Severe postpartum mental illness can be tragic: the leading cause of pregnancy-related deaths is mental health conditions (22.7%), including from suicides and overdose related to substance use disorders (9). The need for enhanced prevention and treatment for PMADs is therefore urgent. The role of cognition in PMAD etiology is a promising area of investigation into targets for PMAD intervention, considering evidence that important cognitive changes occur during the perinatal period, and given that cognitive and information processing alterations are key features of mood disorders. Here we review evidence for cognitive plasticity in matrescence, with a particular focus on executive function (EF) given its overlapping significance for adaptation to parenthood and in mood regulation. To bridge what is known about cognitive endophenotypes of depression within and beyond the perinatal period, this review will highlight key findings from current research on cognitive changes in mood disorders across matrescence. Because data in the perinatal period is limited, we will supplement with what is known about cognition in major depressive disorder and extrapolate how the relationship between cognition and mood may present in perinatal depression. We will also explore the emerging concept of “the mental load of motherhood” as a unique stressor that is likely taxing a mother’s cognitive capacity and contributing to vulnerability to mental illness throughout matrescence.

## 2 Neuroplasticity in matrescence

Matrescence is a term coined by anthropologist Dana Raphael in the 1970s to describe the transition to becoming a mother (10). This transformative process of adaptation to the new role and responsibility of caring for young involves profound hormonal, physiological, psychological, and social changes. The hormones of pregnancy, birth, and lactation drive rapid and extreme physiological transformations that are unparalleled across the lifespan (11–14). Alongside these changes, the maternal brain undergoes significant structural and functional neuroplasticity (14). Neurobehavioral plasticity throughout matrescence may usher in adaptive changes to support a mother in her new role as caregiver, such as by priming attention and motivation to be directed towards infant cues, and altered information processing in preparation for new demands and increased mental load of parenting (14). When viewed through this lens, perinatal cognitive and neural plasticity represents an overall adaptation to caregiving experiences that facilitates learning the skills of parenting and coping with a new set of challenging demands (15). These adaptations are underscored by evolutionarily conserved plasticity in brain structures that are part of a global “parental caregiving networking” that support mammalian caregiving, including sensitivity to infant cues and infant-parent biobehavioral synchrony (15).

Foundational studies in rodents have demonstrated significant neural plasticity in the peripartum period including alterations in neurogenesis, neuronal morphology, and synaptic plasticity throughout the brain, particularly within the hippocampus, prefrontal cortex, basolateral amygdala, nucleus accumbens, and hypothalamus (13, 16, 17) A small but growing body of evidence has demonstrated that primiparous women undergo extensive and highly consistent reductions in regional gray matter volumes across pregnancy, with pronounced reductions in the entire brain present in the early postpartum compared with preconception (18). These gray matter reductions persist for at least two years after delivery (18). While the whole brain appears affected, anatomical changes appear especially pronounced in prefrontal regions associated with the Default Mode and Frontoparietal Networks, including the medial frontal cortex, precuneus, posterior cingulate cortex, inferior frontal gyri, and superior temporal sulci (18–20). The first studies to examine brain change underway *during* pregnancy have emerged only recently: data suggests a clear linear decrease in gray matter volume beginning early in pregnancy, with a sudden shift in direction after delivery where volumetric gains are observed (19–21). Evidence so far of this “fine tuning” of the maternal brain across the perinatal period has been interpreted as adaptive for caregiving and other postpartum behaviors, given that the degree of anatomical change is positively associated with measures of postpartum attachment, and that data so far demonstrates a remarkably consistent trajectory of neural anatomical change in healthy women (18, 22). Given the centrality of prefrontal regions to Theory of Mind (ToM) processes and the importance of these abilities in sensitive caregiving, it might be expected that enhanced ToM ability is a cognitive mediator of anatomical change in prefrontal regions with caregiving behavior, though this remains a gap in literature (22, 23). As the neuroscience of matrescence continues to unfold, examination of how neural functional, anatomical changes, cognitive shifts, and mood symptoms interact will lead to insights into how these neurobiological changes may facilitate the transition to parenthood. In particular, studies in large samples examining individual differences in perinatal neuroplasticity are needed to understand how these potentially influence vulnerability to psychiatric disorders (24, 25).

## 3 The link between cognition and emotion

Cognitive function has long been linked to emotional health. The ‘emotional brain’ and the ‘cognitive brain’ cannot be separated, as there is significant overlap and interactive effects among neural networks (26). Executive function (EF) is an umbrella term for higher-order cognitive processes mediated by the frontal cortex that are essential for goal-directed behavior. EF includes attention, planning, cognitive flexibility (shifting between ideas), multi-tasking, problem-solving, cognitive inhibition, abstraction, and working memory (27, 28). EF plays an important role in facilitating emotion regulation via inhibitory control, particularly impulse control in the context of both positive and negative emotion (29). Research has suggested that strong inhibitory control is foundational to effective emotion regulation, which is the ability to modulate emotional responses in a flexible and adaptive way (30). Emotion regulation has been increasingly integrated into models of psychopathology, and difficulties with emotion regulation are a core feature of mood disorders including depression, anxiety, and bipolar disorder (31, 32). Enhancing emotion regulation strategies is a target for clinical intervention, as strategies such as reappraisal and acceptance are significantly positively related with well-being (33).

Human parenting is a complex task and requires high-order executive functioning and effective emotional regulation to facilitate sensitive caregiving and parental coping and adaptation (34, 35). Parenting requires a balance between effectively and promptly attending to and responding to infant cues, while maintaining a regulated state in order to sensitively respond to infant needs (34). Processing of infant distress cues are particularly salient, which are inherently emotionally evocative and draw on emotion regulation capacities of parents (36). Accordingly, new mothers show enhanced neural activation in the emotional regulation and cognitive control circuit, with prefrontal cortex activation in response to infant distress cues serving to regulate amygdala activation in the face of negative infant stimuli (37). Further, effective emotion regulation affects the health and wellbeing of both mother and baby. Children may learn to regulate their emotions by employing comparable regulatory approaches of their parents, which may lead to the transmission of adaptive as well as maladaptive regulation skills (34, 38–40). Given the bidirectional, dyadic nature of maternal-child mental health, this may in turn have a further positive impact on maternal mood as effective child self-regulation lessens parenting burden. Overall, emotion regulation plays an essential role in a mother’s psychological health as she navigates the responsibilities and demands of motherhood.

### 3.1 Cognitive shifts in matrescence

Despite the overall adaptive role of cognitive and behavioral changes across matrescence, subjective pregnancy-related cognitive deficits are common anecdotally, and perpetuate the negative concept of “mommy brain.” (41) Studies using self-report measures have found that between 50% to 80% of pregnant women report subjective cognitive complaints or memory difficulties, measured using brief self-report questionnaire items or qualitative methods (42–46). However, subjective reports of cognitive complaints are often at odds with performance data from objective cognitive measures. Studies that objectively measured memory performance using standardized assessments show only small differences between perinatal and control groups, primarily concentrated on prefrontally-mediated domains such as working memory performance (27, 41, 43, 47–49). These included a broad range of standardized neuropsychological measures commonly used to evaluate EF and cognition, such as the Color-Word Stroop Task, the WAIS-III Digit Symbol Task, the Backward Digit Span Test, and verbal paired associates task (27, 49–52). The small differences demonstrated on objective cognitive measures likely do not translate into clinically meaningful differences in function, but may result in greater effort involved in these cognitive processes, contributing to subjective mental fogginess. Furthermore, it is important to distinguish normative cognitive variations associated with the transition to parenthood from deficits that meet the threshold of clinical concern. Mild, transient cognitive changes typically do not interfere with daily functioning, quality of life, or parenting capacity and therefore likely do not constitute a clinically significant cognitive “deficit” or “impairment” beyond what would be expected in a major life adaptation (53, 54).

While the term “mommy brain” is often associated with memory difficulty, the phenomenon may reflect broader executive function changes, as memory requires executive functions including attention, cognitive flexibility, and the ability to distinguish salient information to remember. However, research on perinatal changes in executive functioning remain limited. One meta-analysis found that in the third trimester of pregnancy, women experience deficits in general cognitive function, memory, and executive function, but not during prior trimesters and in non-pregnant controls (27). The findings in this meta-analysis should be interpreted with caution given the small to moderate effect sizes of the differences, medium to high heterogeneity, and the limited number of longitudinal studies available. While findings indicate noticeable but minor cognitive changes, significant impairments in complex tasks appeared less likely, and performance generally fell within normal ranges (27). Another meta-analysis presented results consistent with this finding, also reporting that differences in cognition associated with pregnancy tend to be small and concentrated on specific tasks that tax executive function (49). Specifically, memory measures that place relatively high demands on executive cognitive control and effortful processing, such as free recall (6 independent studies; total *N* = 419) and executive components of working memory (4 independent studies; total *N* = 211) appeared to be selectively disrupted (49). The decrement was relatively subtle, and the same specific deficits associated with pregnancy were also observed postpartum (49). These findings point to how the perinatal period may be a time of heightened sensitivity to demands on executive cognitive control, or a heightened mental load. However, studies included in this meta-analysis exhibited high heterogeneity, with much of the variability attributed to the included measures tapping different aspects of memory, as well as sampling error from the small sample sizes in individual studies. There also remain gaps in knowledge about the role of parity and whether the observed cognitive changes mirror those seen in neurological or psychiatric disorders. Further longitudinal research with consistent methodologies is needed to clarify the progression and real-life impact of these cognitive changes during pregnancy.

Executive functioning in the perinatal period deserves particular attention due to its crucial role in parenting, its association with psychiatric disorders, and its vulnerability to environmental demands. Notably, in a small cross-sectional study (*N* = 85), it was found that when objective memory testing was conducted in a home environment, pregnant women performed worse on prospective memory tasks than non-pregnant controls (55). This finding highlights the impact of the home environment on executive functioning, as prospective memory is a process that relies heavily on executive cognitive control and refers to the ability to remember and execute intended actions at a later time. The finding that executive functioning may be worse in the home environment is likely due to the presence of competing demands and distractions that are absent in a controlled laboratory environment. Though majority of the pregnant women in this study were primiparous, mothers with other young children at home face even more distractions, increased responsibilities, and a generally higher mental load. Thus, the heightened executive function demands of parenting, especially in the home environment, may contribute to cognitive challenges. In fact, mothers have reported that interruptions, cognitive overload, and newfound anxieties are core components of the experience of “mommy brain.” (56) As such, the concept of the mental load of motherhood is important to consider as either a cause or consequence of the overloaded “mommy brain.” Executive function may represent a nexus between neurobiological changes and social determinants of maternal mental health.

### 3.2 Executive function in postpartum depression

Given the role of EF in adapting to the demands of parenthood, changes in EF may be particularly significant in the context of postpartum depression (PPD), where cognitive and emotional disruptions impact the responsibilities of caregiving. PPD falls within the umbrella of perinatal depression, which is defined in the DSM-V as a major depressive disorder that occurs during pregnancy or within four weeks after giving birth (57). However, it has been recommended that this criterion be extended to 6 months after delivery (58). Deficits in executive function in the postpartum period can have significant impact as they impair a mother’s ability to care for both herself and her child. This can compound challenges associated with mental illness and have enduring effects on mother–infant interactions and child development (6, 7). Despite the prevalence and impact of perinatal mental illness on both mother and child, understanding of the neural mechanisms underpinning peripartum depression and associated cognitive endophenotypes remains limited.

A small but growing body of research highlights a unique neurobiology of perinatal mental illness that involves an interplay of reproductive hormones, oxytocin, inflammation, the kynurenine pathway, genetic factors, and stress (59). Neuroimaging studies indicate differences between women with PPD and controls in functional connectivity in regions of the brain that overlap with the “caregiving network” (60). Functional neuroimaging studies have shown PPD to be associated with altered activity patterns both at rest and in response to specific emotional cues in brain regions involved in executive functioning, including the dorsal medial prefrontal cortex, orbital frontal cortex, and superior frontal gyrus (61, 62). These regions are critical for self-regulation, decision-making, empathy and emotional processing, functions necessary for managing the complex demands of motherhood. While neuroimaging studies have identified functional alternations in brain regions involved in EF among women with PPD, it remains unclear how these neural changes translate to measurable deficits in neuropsychological performance, underscoring the need for studies that directly link brain function to executive functioning task outcomes.

Few studies have directly examined associations between PPD and EF. One cross-sectional study, involving a larger sample than previous in the literature (*N* = 395), assessed working and short-term memories in mothers and fathers and found that both mothers and fathers with postpartum depression performed worse in a working memory test (63). Notably, the participants were assessed in home visits, where cognitive demands of the home environment may have amplified any cognitive vulnerability associated with PPD. This approach enhances ecological validity and highlights how a high mental load in parenthood may be especially difficult for individuals struggling with PPD. Pregnancy-related depression and anxiety symptoms in mid-pregnancy have been associated with significantly more errors in a visuospatial working memory and executive function task, compared to performance in mothers with low psychiatric symptom levels (64). This finding suggests that EF impairments associated with perinatal depression could impact a mother’s ability to manage the complex demands of caregiving, potentially leading to further stress and worsening mood symptoms. Another cross-sectional study compared executive functioning in third-trimester pregnant women with depressive symptoms to those without depressive symptoms, finding worse cognitive inhibition performance in women with depressive symptoms (65). This finding has implications for emotion regulation, as cognitive inhibition allows for suppression of and a shifting away from negative thoughts in order to regulate emotions. As such, there may be a positive feedback loop between depressive symptoms, impaired cognitive inhibition, and further affective dysregulation. It remains unknown whether EF decrements serve as a risk factor for PPD or whether the cognitive demands of early parenthood exacerbate existing vulnerabilities.

There remains an opportunity to further investigate the neurocognitive profile of PPD, drawing on approaches from studies of cognitive endophenotypes in non-pregnant populations. Cognitive endophenotypes are measurable aspects of cognition that serve as intermediate markers linking risk factors to psychiatric conditions or may represent a prodromal state. For example, a prominent endophenotype in bipolar disorder seems to be a response inhibition deficit, a potential marker of ventral prefrontal dysfunction (66). In major depressive disorder (MDD), impairments in executive cognitive function including selecting strategies, planning, and monitoring performance) are seen, though are not specific for MDD (67). These cognitive endophenotypes may offer valuable insight into how shifts in executive function and related neural mechanisms may impact or predict mood disorders in the perinatal period.

More research is needed to better understand cognitive endophenotypes for PMADs. Because research into cognitive endophenotypes in the perinatal period is relatively scarce, understanding what it known about neuropsychological deficits, specifically executive functioning in MDD outside of pregnancy, may provide important insights into perinatal cognitive endophenotypes.

### 3.3 Executive functioning in major depressive disorder

MDD is associated with cognitive deficits, particularly in EF, memory, and attention. A cognitive model of depression suggest that impaired cognition plays a crucial role in the onset and maintenance of depression by reinforcing negative information processing biases (68). The cognitive neuropsychological model of depression also suggests that treatments for MDD exert their beneficial effects by alleviating these biases (69). Functional neuroimaging studies have linked executive dysfunction in MDD to altered activity in prefrontal executive networks during common cognitive performance tasks (70–72). Specifically, there is evidence for alterations in key emotion regulatory regions, including abnormally increased activity in the dorsolateral (dlPFC) and ventrolateral prefrontal cortex (vlPFC) and decreased activity in the dorsal anterior cingulate cortex (dACC) (72). Therefore, even in the absence of meaningful differences in performance, there may be aberrant patterns of brain activation during executive tasks among depressed populations. Whether the same is true in PPD specifically is not clear.

Cognitive deficits are a well-documented aspect of MDD, affecting executive function, working memory, attention, and learning (73). One meta-analysis demonstrated that MDD is consistently linked to worse performance on neuropsychological measures of EF, with moderate to substantial effect sizes (74). There is strong evidence for impairment in processing speed, learning, and memory in an acute depressive episode (73). These deficits have been found to be more pronounced in individuals experiencing more severe symptoms and those on psychotropic medications (74). Depressed patients require greater cognitive effort and longer cognitive processing time to execute executive functioning tasks (75). Impaired cognition occurs in around two-thirds of depressed patients (76) and persists beyond acute episodes. Individuals with MDD, even in remission, exhibited moderate deficits in EF and attention, with smaller but persistent impairments in memory (77). About one-third to one-half of remitted depressed patients are thought to be affected by cognitive deficits (78, 79). Evidence from another meta-analysis indicates that the deficits that remain in remission are more mild than in acute episodes, and include working memory, attention, and learning and memory functions (73). Cognitive impairments in depression contribute to its chronicity, treatment resistance, and functional burden (69, 80). While more research is needed to determine the casual relationship between MDD and cognitive dysfunction (81), it presents a promising paradigm that could be applied to PPD.

### 3.4 Understanding the cognitive burden of motherhood: Interactions between mental load and executive function

The concept of mental load captures the cognitive demands placed on an individual when performing tasks or processing information (82–85). It encompasses the mental effort and resources required to manage and complete various cognitive activities and can be seen as the “cost” of mental labor on the limited mental capacity of an individual (84). Mental overload has been defined as a mismatch between task demands and available resources (85). Findings in domains outside of parenting emphasize the role of executive function in managing and perceiving mental load and hold implications for how cognitive resources are allocated in complex, multitasking environments (86). In parenting, if the demands of this cognitive and emotional labor exceed a parent’s ability to cope, parents may experience cognitive overwhelm, emotional strain, reduced cognitive and emotional resources for bonding, and taxed executive functions. A limiting factor in this field is the current lack of validated instruments for measuring mental load of parenting, in part due to the complexity of measuring this phenomenon outside of constrained laboratory-based tasks, where the concept originated (83, 87–89). Despite this challenge, an integration of cognitive and social dimensions of mental load provide a useful framework for understanding how matrescence may tax cognitive resources. The term “The Mental Load of Motherhood” (MLM) can be understood as the overall mental load required to parent. MLM involves monitoring tasks that need to be completed while simultaneously managing the past, present, and future emotions of each family member individually, as well as the collective family (83). MLM encompasses a component of cognitive labor, emotional labor, and is thought to be impacted by psychosocial stressors (87). Beyond the hands-on tasks of caregiving, MLM entails the often invisible work of managing household responsibilities, such as accountability for the task outcomes, anticipating and planning for the family’s long-term needs, making decisions, and monitoring progress, which is mentally taxing yet often invisible to both cognitive laborers and their partners (88). A phenomenological analysis of seven focus groups interviews with mothers of young children further delineated the definition of a mothers’ mental labor, grounded in lived experience of mothers (90). The mental labor of motherhood emerged as a set of six cognitive activities aimed at accomplishing family goals: planning and strategizing, monitoring and anticipating needs, meta-parenting (thinking and reflection about parenting), knowing (learning and remembering), managerial thinking (including delegating and instructing), and self-regulation (90).

In addition to cognitive labor, within the mental load of motherhood also lies emotional labor, which is defined as the work of managing one’s own emotions and those of others. In the setting of family life, emotional labor is the work of anticipating, thinking, and caring about family needs and feelings (83). Emotion regulation and executive functioning are important skills in this work, as the there is a need to regulate one’s own emotions in order to respond to children with patience and sensitivity, and there is often a need to suppress one’s own thoughts and emotions to prioritize caregiving. Thus, emotional labor is a core component of MLM, requiring continuous emotional attunement and self-regulation in service of the family’s emotional well-being.

Psychosocial and structural factors play a critical role in the increased modern day mental load of motherhood. Firstly, despite improvements in domestic labor inequality, disparities remain in the division of domestic work. Mothers often bear the bulk of the physical household labor, with an even more disproportionate share of the cognitive load of household responsibilities (88, 89, 91). Social determinants of health further exacerbate modern stressors for subgroups of women facing additional adversities. Factors such as cultural values, minority stress, financial burden, parental leave policies, the isolation of nuclear family structures, the stress of single parenting, and other interpersonal and family dynamics contribute to MLM (87, 90). Emerging literature from neuroimaging studies of the maternal brain suggest potential interactive effects of these factors with cognitive load on maternal brain functioning, which may present a neurocognitive mediator of heightened risk for postpartum mental health problems. Evidence reviewed in Kim et al. (2021) demonstrates overall that a larger number of socioeconomic, physical, environmental, and psychosocial stressors is associated with altered maternal brain activation in areas important for emotional and cognitive empathy in response to infant cues, which in turn impacts a mother’s capacity for sensitive caregiving, decision-making, and emotion regulation (37, 87, 92). More optimistically, two separate interventions aimed at improving emotional regulation skills and inhibitory control among low income women have demonstrated effectiveness in improving parenting skills and maternal wellbeing, highlighting EF as a potential modifiable intervention target for at-risk groups (93, 94). Social policies can also have positive impacts: for example, policies supporting mothers through paid maternity leave are associated with reduced maternal stress and improved familial mental health (95). Still, further research is needed to determine which familial, institutional, and social factors surrounding modern day motherhood contribute most to the mental load burden, and how MLM interacts with broader social determinants of health.

Neuroscience has scarcely accounted for the mental load of motherhood. It remains an unmeasured but potentially significant factor in understanding perinatal cognitive and mood changes. Cognitive adaptations to parenting and managing MLM place demands on executive functioning include cognitive flexibility, emotional regulation, theory of mind, decision making, goal-directed behavior, and reward sensitivity (18, 61, 96). Executive functioning is core to daily functioning, well-being, the regulation of cognitive processes that impact the quality of life, as well as multiple aspects of childcare, including anticipating, identifying, scheduling, planning, organizing, deciding, and ultimately ensuring that household and childcare-related tasks occur.

It is unclear how MLM and EF intersect to contribute to mood and subjective cognition in the peripartum (41, 97). Existing research exploring the etiologies of pregnancy-related memory difficulties have attributed this to complex hormonal changes, changes in neurotransmitters, changes in the chronological age of circulating erythrocytes, mood alteration, cultural stereotypes, and lifestyle factors (41, 46, 49, 98–100). The role of MLM in these subjective cognitive changes has not been well accounted for, though it is an important factor in maternal well-being. Cognitive labor has been linked to heightened risks of maternal depression, stress, burnout, and relationship strain (89). The cognitive load of parenting seems to be particularly taxing, as evidence suggests that sharing cognitive labor with a partner is more effective at reducing maternal stress than merely delegating physical tasks like diaper changing (101, 102). Thus, the growing burden of the mental load of motherhood is likely a key factor driving vulnerability to mental illness across matrescence (87). The complex phenomenon of the mental load of motherhood impacts the majority of mothers and must be measured and integrated into maternal mental health research (87).

## 4 Discussion

### 4.1 Summary of findings

The evidence reviewed highlights the strong association between MDD and executive function impairments, reinforcing cognitive theories that suggest these deficits contribute to the onset and maintenance of depression. The persistence of EF deficits even in remitted individuals underscores their relevance as both a state and trait marker of depression. Given the cognitive and emotional regulation demands of the perinatal period, such findings provide a valuable framework for understanding similar impairments in postpartum depression. The mental load of motherhood, coupled with neuroplastic changes in the perinatal brain, may further exacerbate EF vulnerabilities during this time and heighten risk for PMADs.

Although the literature on MDD and EF is extensive, evidence for EF changes in PPD remains more limited. Current findings suggest that PPD may also be associated with decrements in EF that are essential for managing the demands of parenting, including worse cognitive inhibition, working memory, and short-term memory (63–65). These EF decrements may contribute to difficulties in caregiving, potentially increasing maternal stress and worsening mood symptoms. However, research on the relationship between PPD and executive function remains limited, with a predominance of cross-sectional studies.

Structural brain changes during matrescence more broadly, in frontal cortical regions that are strongly associated with executive functioning, reinforce a potential role of EF in both adaptation to parenting and vulnerability to mental illness. Despite the common experience of “mommy brain, “ existing research has shown that cognitive changes across the perinatal period are relatively subtle and primarily emerge during cognitive tasks that place high demand on executive function (27, 41, 43, 47–49). Although a meta-analysis supports this finding, significant heterogeneity across studies and inconsistent results limit definitive conclusions.

### 4.2 Limitations and gaps in the literature

Until recently, there has been a striking lack of research on the maternal brain across the perinatal period. This gap reflects broader systemic issues in science, where women’s health research has historically been underfunded and overlooked, hindering progress in understanding and improving maternal mental health (103). Inconsistent findings across existing studies may stem from variability in cognitive tasks, timing of assessments, and the heterogeneity of perinatal populations. Longitudinal research, with more consistent methodologies such as cognitive tasks and time points, is essential for capturing the dynamic cognitive and emotional changes across the perinatal period, and for identifying early markers of risk. There is also growing recognition that pregnant women and mothers with PPD are not a homogenous group. A recent systematic review identified four major symptom trajectories within PPD, highlighting the need to move beyond static models of perinatal depression (104). Longitudinal designs, by studying the same people over time, are essential for distinguishing true cognitive shifts from individual variability.

Possible parallels between EF impairments in MDD and those reported in postpartum depression invites further investigation into whether cognitive difficulties during the perinatal period might reflect maladaptive changes associated with the onset of mood disorders versus expected neuroplastic and cognitive adaptations to the demands of motherhood. Cognitive resource allocation theory suggests that during the perinatal period, neural resources may be redistributed from executive function domains toward enhanced social-cognitive capacities, such as emotional processing and theory of mind, to enhance caregiving (105). This adaptive perspective challenges deficit-based perspectives and highlights the need for research that can differentiate between maladaptive impairment and adaptive reorganization supporting the transition to parenthood.

### 4.3 Future directions and recommendations

To advance understanding of EF changes across the perinatal period, several research priorities emerge. Longitudinal designs are needed to understand normative cognitive trajectories over time, better control for individual variability, and distinguish between transient shifts and lasting impairments. Future studies should also adopt ecologically valid assessments, including parenting-relevant stimuli and home-based cognitive tasks to better capture possible adaptive enhancements as well as the impact of the real-world cognitive demands of motherhood on EF and memory performance. Future research should also study maternal cognitive and mental health in context. The early postpartum time frame has a number of confounding physiological effects, including sleep deprivation, stress, fluctuating neurohormones, baby blues, parity, and the often overlooked emotional and cognitive labor of motherhood, all of which interact with cognitive function and mental health outcomes (106). There is a need to better understand how the cognitive and emotional labor of parenting might interact with neural plasticity, executive function, and ultimately risk of psychiatric illness.

Reconceptualizing “mommy brain” as a potential form of cognitive adaptation, rather than solely a deficit may help shift narratives and advance science, as cognitive resources during matrescence may not be diminished but reprioritized (107). A nuanced reconceptualization of “mommy brain” within the context of the modern-day mental load of motherhood and with respect to neural adaptations to parenting will integrate neurobiological perspectives and social determinants of health, ultimately advancing a more comprehensive approach to maternal mental health.

## 5 Conclusion

The perinatal period represents a time of profound neurobiological, cognitive, and emotional change. While these adaptations support the transition to motherhood, they also may heighten vulnerability to mental health challenges, including PPD. Executive function, as a critical cognitive domain, plays a central role in managing the mental load of motherhood and has also been implicated in depressive disorders. Understanding normative EF changes during this period is essential for better understanding the relationship between EF, perinatal depression, and the mental load of motherhood. Consideration for these cognitive, neurobiological, and psychosocial factors of matrescence is critical for addressing maternal mental health and developing interventions that support parental well-being.
